# The Effectiveness of Physiotherapy Rehabilitation in an Adult With Multiple Joint Fractures - A Case Report

**DOI:** 10.7759/cureus.63654

**Published:** 2024-07-02

**Authors:** Anjali V Nawkhare, Mitushi Deshmukh, Sakshi Padmawar

**Affiliations:** 1 Department of Musculoskeletal Physiotherapy, Ravi Nair Physiotherapy College, Datta Meghe Institute Of Higher Education and Research, Wardha, IND

**Keywords:** physiotherapy, range of motion, muscle strength, multiple joint fractures, rehabilitation

## Abstract

This case report details the rehabilitation procedure for a 35-year-old man involved in a vehicle accident that resulted in multiple joint fractures. The patient had fractures to the proximal tibia, left bimalleolar, posterior malleolus, fourth and fifth metacarpal heads, and second and third proximal phalanges. After open reduction and internal fixation surgery, an 8-12 week physical treatment regimen was put into place. Exercises for both passive and active range of motion, isometric and progressive resistance training, and gait training were provided. The rehabilitation goals were pain relief, increased range of motion, muscle strength, flexibility, endurance, and functional independence. Pain levels, range of motion, muscle strength, and general function all significantly improved between pre- and post-rehabilitation evaluations. Early mobilization and structured physical therapy were crucial in achieving these outcomes, highlighting the importance of tailored rehabilitation protocols for post-operative recovery.

## Introduction

Multiple joint fractures involve two or more bone fractures on the different joints of the human body. The ankle is one of the most commonly fractured lower limbs, accounting for approximately 9% of all cases, with an annual incidence of 107-187 per thousand people [1]. The most common fractures in these age groups occur in men under 50 years of age and in women over 50 years of age. Among the most common causes of ankle fractures are motor vehicle accidents, twisting injuries, and falls [2]. The tibia is the most commonly broken of all the long bones in the human body, and it is generally broken as a result of high-energy trauma, such as being involved in a car accident or falling from a great height [3]. In approximately 88% of all metacarpal fractures, the fifth finger is the most commonly fractured. Eightteen to 44% of all hand fractures are metacarpal fractures [4]. The type and severity of the trauma, the integrity of the fracture fixation, and biological processes, particularly immunological and developmental processes related to skeletal ontology, all affect how quickly a fracture heals [5]. As the incidence of fractures among the senior population rises, so does the illness load. The senior population finds it challenging to follow restrictions on weight bearing, and being immobile comes with serious hazards and increased morbidity [6]. In proximal tibia fracture tibia, a more complicated and multi-fragmented fracture pattern may be the result of high-energy trauma, which mainly affects young people. However, in elderly individuals with lower bone density, a low-energy mechanism may result in a pure depression fracture. Preoperative classification of these fractures with the Muller AO Classification, Schatzker classification, or cutting-edge computed tomography (CT) based techniques aids in understanding the fracture pattern and aids in selecting the surgical approach and treatment plan based on estimated bone mineral density and the unique medical histories of each patient [7].

Thirty to 50% of all hand fractures are metacarpal fractures. The variety and complexity of human hand movements are made possible by the biomechanical interactions between the soft tissues of the hand. Only a sturdy skeleton structure can support the combined action of these mutually beneficial forces. The typical activities of the soft tissue result in deforming postures when the structure is violated by a fracture, which is detrimental to effective fracture therapy [8]. Orthopedic physicians treat bimalleolar ankle fractures more frequently than any other type of injury. Ankle fractures with bimalleolar bones are typically brought on by twisting injuries with numerous force mechanisms. The ligaments surrounding the medial and lateral malleoli give the ankle joint support. An intra-articular injury is a bimalleolar fracture. Normalizing anatomy is the major objective of treating these fractures. The surgical technique restores the joint's anatomy and contact-loading properties [9].

In this case, a 35-year-old male had a history of road traffic accidents (RTA). The patient came with a complaint of wrist pain and leg pain on the left side for six days and inability to walk properly for six days. Pain and swelling were progressively in nature. On radiological investigation, the patient was diagnosed with multiple joint fractures. Pain and swelling were progressively in nature after the patient was managed operatively with open reduction and internal fixation. After that, postoperative physiotherapy treatment was started on day one, and the physiotherapy protocol was 8-12 weeks. According to the level of stability attained by internal fixation, bone mineral density, and other patient-specific characteristics, rehabilitation procedures should be carefully established (age, compliance, mobility). Early functional mobilization is crucial in rehabilitation to prevent stiffness [7]. The aim of physiotherapy is to reduce pain, increase and maintain a range of motion, improve and maintain strength in the muscle of the metacarpophalangeal (MCP) joint, knee, and ankle joints, and improve flexibility and endurance as well as improve and maintain functional independence.

## Case presentation

Patient information

A 35-year-old man was apparently alright before 23/10/22. He lives in Dhapki village, and he is an astrologist. Hand dominance is right. He was in a road traffic accident, a head-on collision on 17/10/22 at Bilaspur. The patient was unable to walk immediately after the incident. Then, the patient's relative brought the patient to a nearby hospital in Bilaspur, where medication was given, and the dressing was done and managed conservatively. Pain was relieved by medication. Then, the patient came to a super-specialty hospital on 23/10/22 with complaints of wrist pain and leg pain on the left side for six days and inability to walk properly for six days. Pain and swelling were progressive in nature. Then, an orthopedic doctor prescribed medication and further investigation was done. Radiological investigation diagnosed a left-side bimalleolar fracture, posterior malleolus fracture, proximal tibia fracture, fourth and fifth metacarpal head fracture, and second and third proximal phalanx fracture. After that, the patient was referred to a musculoskeletal physiotherapy unit for further physiotherapy management.

Clinical findings

The patient was cooperative, conscious, and well-oriented to time, place, and person. In a supine position with the shoulder and the anterior superior iliac spine (ASIS), both at the same level, and the left leg was elevated. Upon physical examination, pulse rate was 77 beats/min, respiratory rate was 19 breaths/min, and blood pressure was 120/80 mmHg. On observation, the general condition of the patient is fairly built. A plaster cast is present over the left knee joint to the ankle joint and the fourth and fifth fingers to the elbow joint. On palpation, swelling and redness over the left ankle joint, lateral condyle, and metacarpal joint. Grade 2 tenderness was present over the anterolateral aspect of the lateral condyle, ankle joint, and fourth and fifth metacarpal joint. Hamstring tightness was present on the right lower limb.

Pre-rehab Visual Analogue Scale (VAS) was 4.5/10 at rest and 7.5/10 on slight movement, and post-rehab VAS was 2.5/10 at rest and 2.5/10 on slight movement. For motor examination, the goniometer was used to assess the range of motion (ROM), and the results are shown in Table 1 as being within normal limits. Table 2 shows the results of the Manual Muscle Testing (MMT) evaluation.

**Table 1:** Range of motion assessment MCP - metacarpophalangeal joint; PIP - proximal interphalangeal joint

Joint (Left side)	Pre-rehabilitation	Post-rehabilitation
MCP fourth and fifth flexion	0°-20°	0°-75°
MCP fourth and fifthextension	0°-8°	0°-15°
MCP fourth and fifth abduction	0°-8°	0°-18°
PIP flexion	0°-75°	0°-95°
Wrist flexion	0°-78°	0°-78°
Wrist extension	0°-70°	0°-70°
Ulnar deviation	0°-30°	0°-30°
Radial deviation	0°-20°	0°-20°
Knee flexion	0°	0°-135°
Ankle dorsiflexion	0°	0°-48°
Ankle plantarflexion	0°	0°-17°
Ankle inversion	0°	0°-13°
Ankle eversion	0°	0°-28°

**Table 2:** Muscle Manual Testing

Joint (left side)	Pre-rehabilitation	Post-rehabilitation
Wrist flexors	4/5	5/5
Wrist extensors	4/5	5/5
Knee flexors	3/5	4/5
Ankle plantar-flexors	2/5	4/5
Ankle dorsiflexors	2/5	4/5
Ankle invertors	2/5	4/5
Ankle evertors	2/5	4/5

Investigations

**Figure 1:**
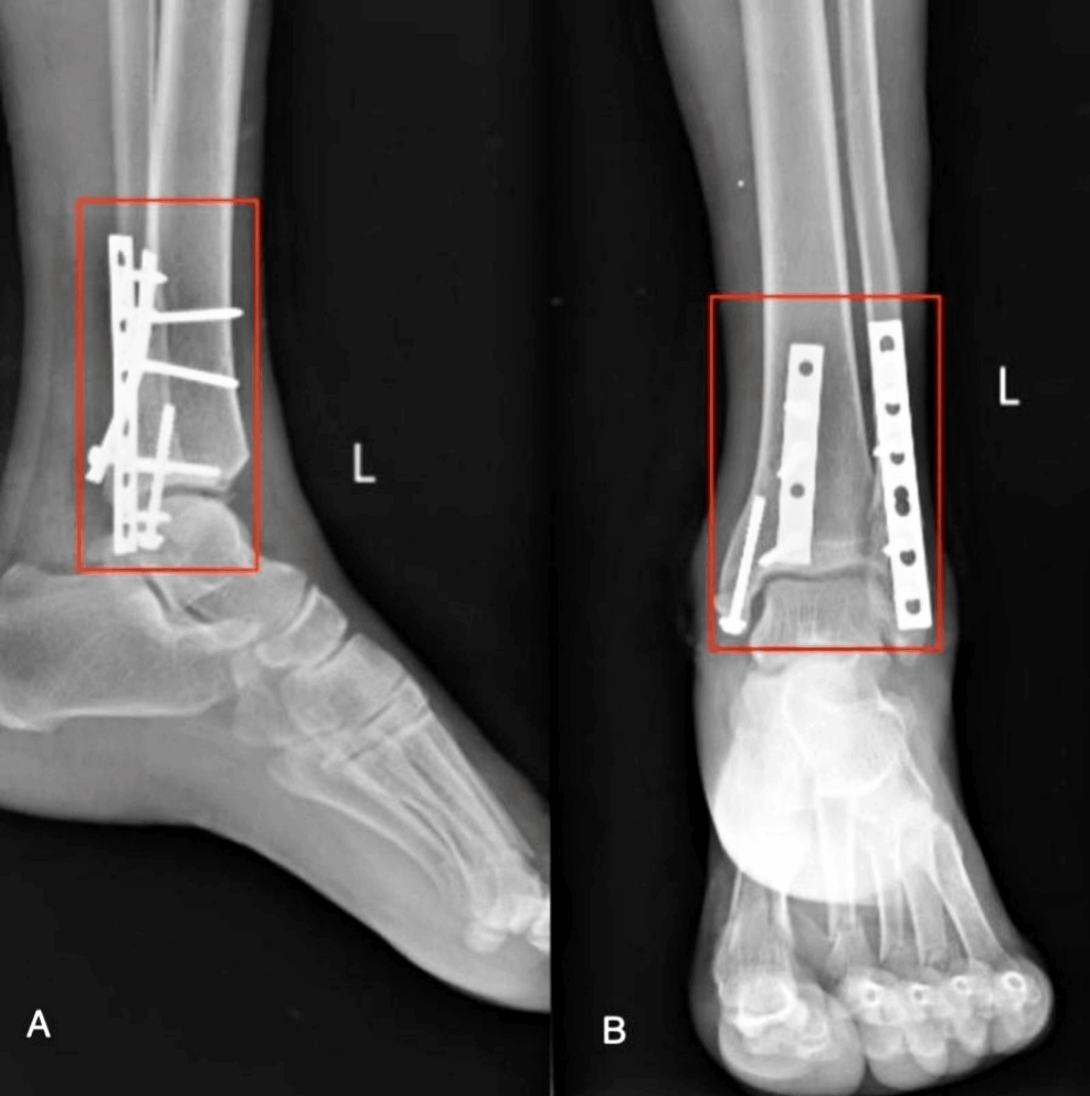
Postoperative X-ray of the left ankle joint AP view AP - antero-posterior A) Semi-tubular plate 6 hole, cortical screw; B) Semi-tubular plate 8 hole, cortical screw

**Figure 2:**
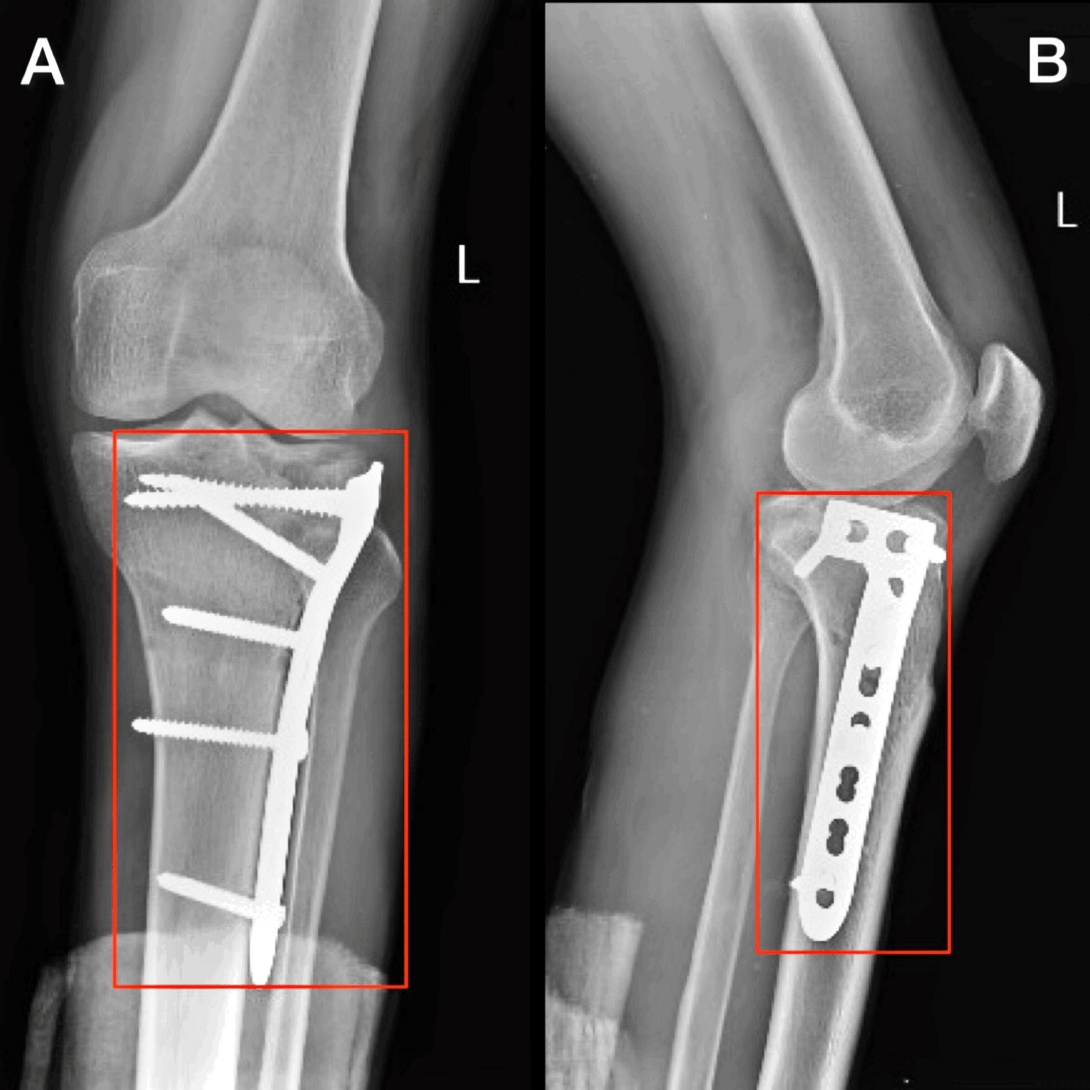
Postoperative X-ray of the left knee joint AP - antero-posterior A: AP view of knee joint showing cancellous screw and locking screw; B) Lateral view of ankle joint showing L-butress plate

**Figure 3:**
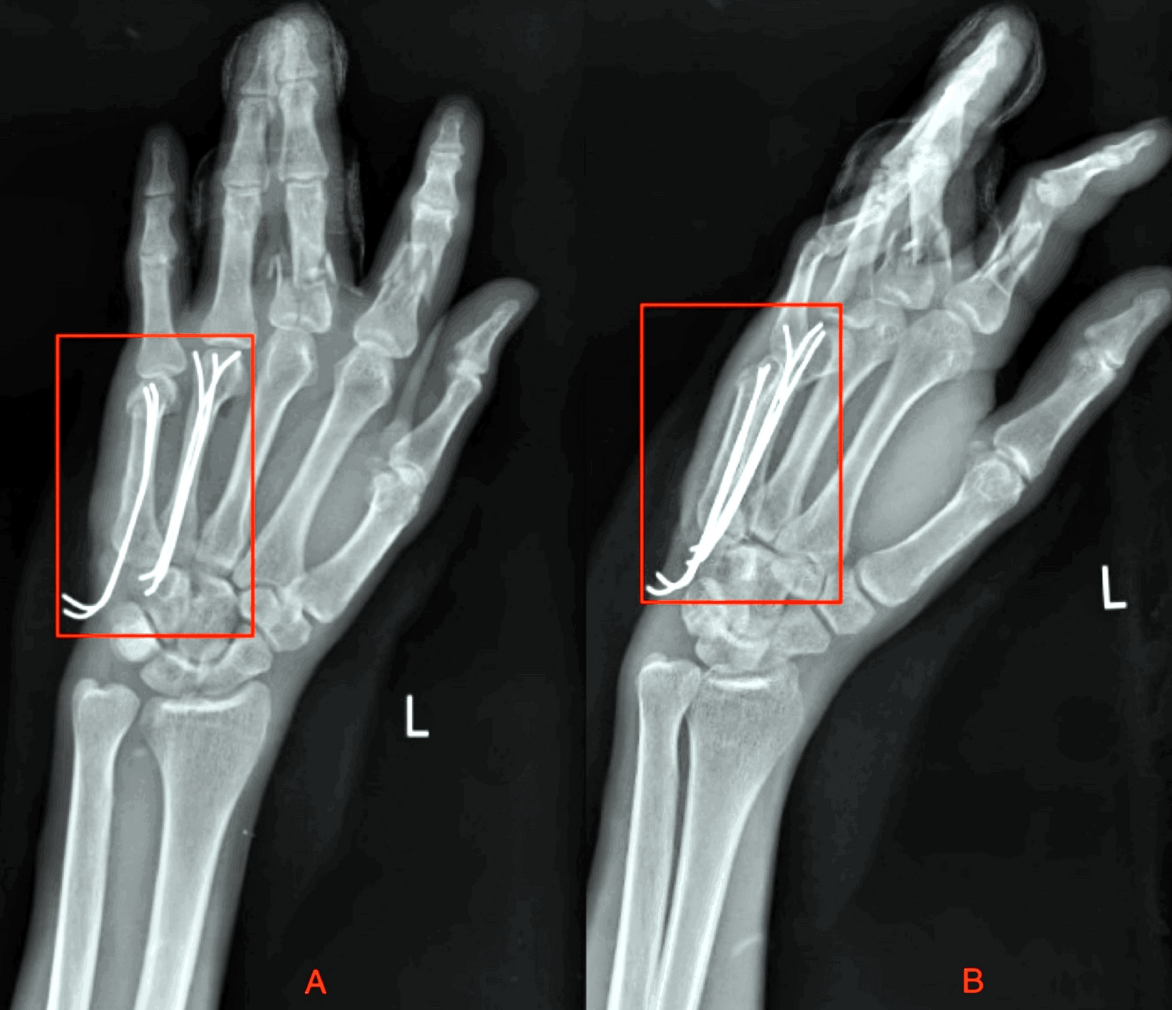
Post-operative X-ray of the left hand A) Antero-posterior view; B) Lateral view

X-ray was done post-operatively. The patient has undergone an operation that is open reduction and internal fixation with plate osteosynthesis for bimalleolus fracture, posterior malleolus fracture, and proximal tibia is shown in Figure 1 and Figure 2, as well as open reduction and internal fixation for fourth and fifth metacarpal head fracture and second and third proximal phalanx fracture shown Figure 3.

Physiotherapy management

**Figure 4:**
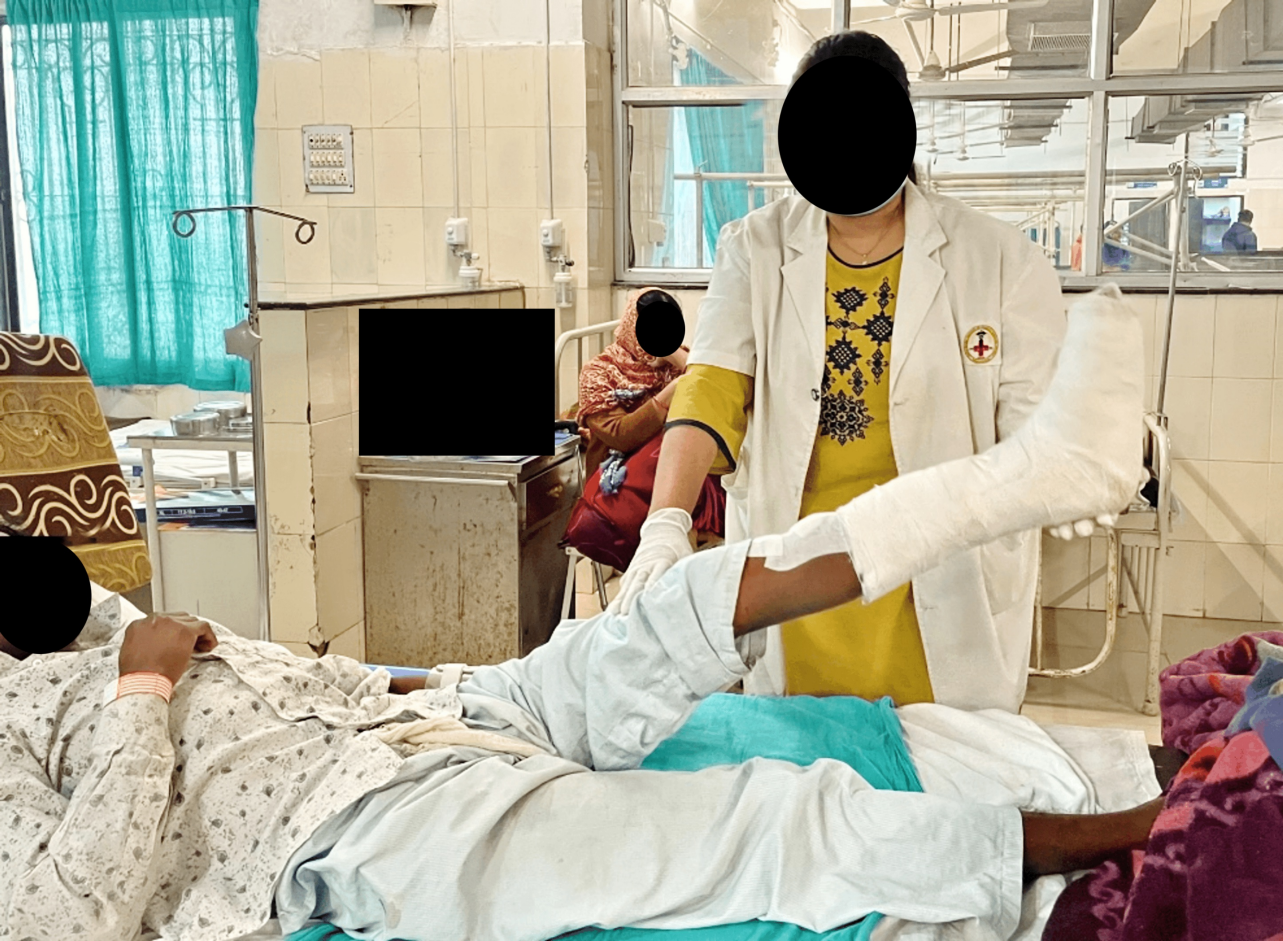
Patient performing assisted SLR SLR - straight leg raise

**Figure 5:**
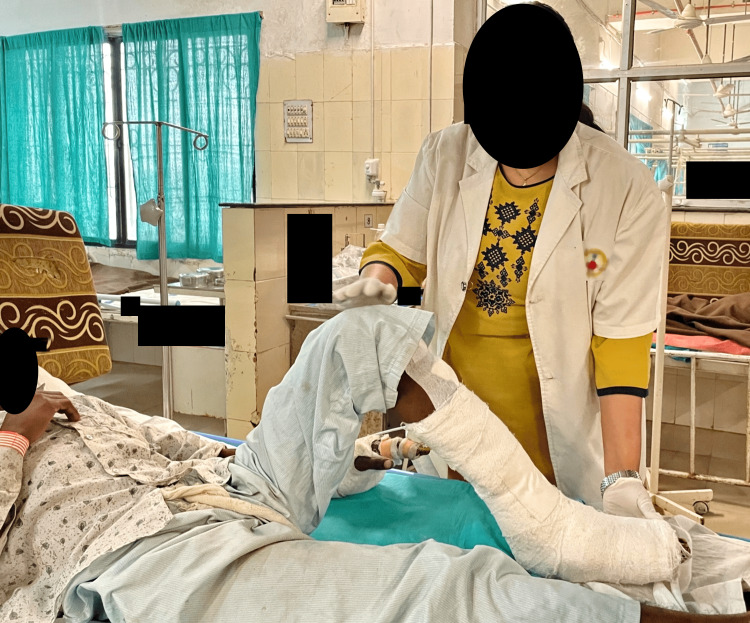
Patient performing active knee flexion exercise

Physiotherapy rehabilitation protocol for this multiple joint fractures is 8 to 12 weeks and divided into four phases. The fracture of the fourth and fifth metacarpal head and second and third proximal phalanx fracture rehabilitation protocol was four to eight weeks (Tables 3, 4), as well as left-side bimalleolar fracture, posterior malleolus fracture, proximal tibia fracture rehabilitation protocol, which was 8 to 12 weeks (Table 3, 5). Precautions taken in phase 1 were no strengthening exercise and no active range of motion (ROM) for the affected side extremity. Figure 4 and Figure 5 show the patient receiving physiotherapy treatment.

**Table 3:** Physiotherapy management phase-wise for the upper and lower limbs ROM - range of motion

Goal	Treatment	Phase 1	Rationale
Patient education	Patient education	Educating the patient and family about their condition and how physiotherapy helps to regain their normal ROM and strengthen	Patient and their family were educated about the importance of physiotherapy
Reducing pain and tenderness	Cryotherapy	10 minute	Vasoconstriction to reduce inflammation and swelling

**Table 4:** Upper limb physiotherapy management phase-wise MCP - metacarpophalangeal joint; ROM - range of motion; MET - muscle energy technique; PIR - post-isometric relaxation

Goals	Treatment	Phase 1 (day 1-1 week)	Phase 2 (2-3 weeks)	Phase 3 (4-8 weeks)	Rationale
For upper limb and MCP joints. To increase and maintain ROM MCP joints.	Active range of motion exercise for elbow and shoulder	10 repetitions 1 set	-	-	Enhancing the motion of a particular joint. The arrangement of the bone surfaces within the joint, the joint capsule, as well as the muscles, tendons, and ligaments that act on the joint all influence this motion
	Active range of motion exercise for metacarpal joint (MCP)	-	10 repetitions 1 set	10 repetitions 2 set	Restore normal and increase as well as maintain joint motion
To improve and maintain muscle strength.	Isokinetic exercise for deltoid, biceps, triceps and quadriceps	10 repetitions 1 set	-	-	To maintain strength for upper limb muscle
	Isometric exercise for wrist flexors, extensors	-	10 repetitions 1 set	10 repetitions 2set	To increase strength and maintain strength of wrist muscles
	MET and PIR for wrist extensors	-	-	10 repetitions 1 set	Lengthen shortened muscles, increase joint range of motion
	Resistance exercise squeezing ball	-	10 repetitions 1 set	10 repetition 2 set	To increase and maintaining muscle strength MCP and PIP joint
	Progressive resistance exercise for hand muscle using weight cuff	-	10 repetitions 1 set	10 repetitions 1 set	To maintain muscle strength

**Table 5:** Lower limb physiotherapy management phase-wise MCP - metacarpophalangeal joint; SLR - straight leg raise; ROM - range of motion, MET - muscle energy technique; PIR - post-isometric relaxation

Goals	Treatment	Phase 1 (day1-1 week)	Phase 2 (2-3 weeks)	Phase 3 (4-8 weeks)	Phase 4 (4-8 weeks)	Rationale
To increase and maintain ROM	Active range of motion exercise for metatarsophalangeal joint	10 repetitions 1 set	10 repetitions 2 set	10 repetitions 2 set	-	Enhancing the motion of a particular joint. The arrangement of the bone surfaces within the joint, the joint capsule, as well as the muscles, tendons, and ligaments that act on the joint all influence this motion.
	Assisted straight leg raise		10 repetitions 1 set	-	-	To increase the range of motion
	Straight leg raise		-	10 repetitions 1 set	-	To maintain normal ROM
	Active range of motion exercise for knee joint		10 repetitions 1 set	-	-	To increase the range of motion of the knee joint
	Active range of motion exercise for ankle joint	-	-	10 repetitions 1 set	10 repetitions 2 set	To increase the range of motion of the ankle joint
To increase and maintain muscle strength	Isometric exercise for dorsiflexors and plantar flexors	-	10 repetitions 1 set	10 repetitions 2 set	10 repetitions 2 set	To increase and maintain muscle strength of dorsiflexors
	Isometric exercise for invertors and evertors	-	-	10 repetitions 1set	10 repetitions 2 set	To increase and maintain muscle strength of invertors and evertors
	MET and PIR for dorsiflexors		-	10 repetitions 1 set	10 repetitions 2 set	Lengthen shortened muscles, increase joint range of motion and increase fluid
	Progressive resistance exercise for lower limb using weight cuff	-	-	-	10 repetitions 1 set	To improve muscle strength and power
	Dynamic quads using weight cuff	-	-	-	10 repetitions 1 set	
To improve and maintain gait pattern and balance	Gait training	Non-weight bearing stand pivot transfer	Toe touch to partial weight-bearing	Ambulation with assistive device Partial weight bearing to a 3-point stance.	Ambulation with assistive device 3-point stance to full weight bear.	Strengthen muscles and joints, improve balance and posture, build endurance

Outcome measures

Also, we used clinical outcome measures for improvement that is pre-rehabilitation and post-rehabilitation which is mentation in Table 6.

**Table 6:** Outcome measure using scales

Scales	Pre-rehabilitation	Post-rehabilitation
Lower extremity function scale	55/80	75/80
Upper extremity function scale	40/80	75/80
Quality of life	50	70
Visual Analogue Scale (VAS)	7.5	2.5

## Discussion

Multiple fractures are typically the outcome of high-energy incidents, which frequently happen to younger patients, according to the majority of surgeons. The highest prevalence of multiple fractures in the elderly was shown to be related to high-energy modes of injury, such as falls from heights or downstairs and RTAs (27.3%, 12.5%, and 36%, respectively). The majority of multiple fractures, however, actually happen after low-energy trauma (88.1%), as these kinds of injuries are rare in senior people [10]. It is asserted that the Muscular Energy Technique (MET) can stretch short muscles, expand the joint range of motion, and promote fluid drainage from peripheral locations [11]. Although there are various ways to use the MET to make muscles more extensible [12], in this case, pursuing the rehabilitation regimen helped the patient achieve functional independence and significantly reduced pain and tenderness, improving range of motion, muscle strength, and activities of daily living (ADL).

Pereira et al., in 2020, conducted a systemic review that mentioned advanced technology for hand rehabilitation that is augmented reality (AR) and virtual reality (VR) has been used to improve the rehabilitation process. Haptic gloves with a leap motion controller can be used in a home environment. Technologies like AR and VR can be utilized in conjunction with traditional treatments. The utilization of AR or VR therapies for hand rehabilitation can be advantageous to patients. AR and VR have been used to improve the rehabilitation process [13].

The study conducted by Donohoe et al. in 2020 found that early mobilization and weight bearing after a fracture are major goals of treatment in order to avoid deconditioning and the negative effects of prolonged immobility [6]. Arslan et al. conducted a study in 2015 that concluded the initiation of early ROM soft tissue lesions altered the onset of early knee joint mobility, which enhanced the clinical outcomes. Therefore, in cases like these, meniscus and ligament injuries should be taken into account as prognostic variables, as well as early passive mobilization, have been proven to reduce the incidence of deep vein thrombosis (DVT) and increase knee flexion range of motion during rehabilitation [14]. In the early stages of maintaining and improving knee mobility, electrotherapy modalities, including continuous passive motion, were used to initiate and enhance the knee range of motion [15].

## Conclusions

According to the above case report, it is associated with a surgical approach, and early, structured physical therapy rehabilitation improved the functional goals over time, which is a crucial factor in helping postoperative patients recover successfully. The patient, in this instance, was greatly helped in achieving functional independence by adhering to the organized rehabilitation program. Reduction of discomfort and tenderness, improvement of range of motion, strengthening of muscles, and improvement of activities of daily living (ADLs) were all made possible by the combination of early, comprehensive physiotherapy rehabilitation and surgical intervention. A thorough, progressive physiotherapy approach is essential for managing difficult multiple-joint fractures, as demonstrated by the patient's notable improvement in strength and mobility. The rehabilitation procedure enhanced the patient's general health and quality of life in addition to benefiting in their recovery from injury. The successful recovery of postoperative patients with multiple joint fractures is largely dependent on early mobilization and physiotherapy rehabilitation, as this case study demonstrates.
